# Unleashing the potential of the root hair cell as a single plant cell type model in root systems biology

**DOI:** 10.3389/fpls.2013.00484

**Published:** 2013-11-26

**Authors:** Zhenzhen Qiao, Marc Libault

**Affiliations:** Department of Microbiology and Plant Biology, University of OklahomaNorman, OK, USA

**Keywords:** stress response, root hair cell, single plant cell type, systems biology, ultrasound aeroponic system

## Abstract

Plant root is an organ composed of multiple cell types with different functions. This multicellular complexity limits our understanding of root biology because -omics studies performed at the level of the entire root reflect the average responses of all cells composing the organ. To overcome this difficulty and allow a more comprehensive understanding of root cell biology, an approach is needed that would focus on one single cell type in the plant root. Because of its biological functions (i.e., uptake of water and various nutrients; primary site of infection by nitrogen-fixing bacteria in legumes), the root hair cell is an attractive single cell model to study root cell response to various stresses and treatments. To fully study their biology, we have recently optimized procedures in obtaining root hair cell samples. We culture the plants using an ultrasound aeroponic system maximizing root hair cell density on the entire root systems and allowing the homogeneous treatment of the root system. We then isolate the root hair cells in liquid nitrogen. Isolated root hair yields could be up to 800 to 1000~mg of plant cells from 60 root systems. Using soybean as a model, the purity of the root hair was assessed by comparing the expression level of genes previously identified as soybean root hair specific between preparations of isolated root hair cells and stripped roots, roots devoid in root hairs. Enlarging our tests to include other plant species, our results support the isolation of large quantities of highly purified root hair cells which is compatible with a systems biology approach.

## EXPERIMENTAL OBJECTIVES

Our understanding of root biology (i.e., root development, root cell differentiation and elongation, response to biotic and abiotic stresses) is based on -omic studies performed at the level of the entire root system or specific regions of the root as well as from the identification of mutants showing defects in root development. These mutants were characterized from the model plant *Arabidopsis thaliana* (Benfey et al., 1993; Rogg et al., 2001; Mishra et al., 2009) as well as other plants where genetic tools are well developed [e.g., *Medicago truncatula *(Tadege et al., 2008), *Oryza sativa *(Kurata and Yamazaki, 2006), *Lotus japonicus *(Schauser et al., 1998; Perry et al., 2003)]. These valuable studies led to the identification of important genes and even gene networks controlling plant development and adaptation to stresses (Schiefelbein et al., 2009; Bruex et al., 2012).

To enhance our current understanding of root biology, a systems biology approach is needed to take advantage of the recent improvements in technologies such as mass spectrometry and high-throughput sequencing. One challenge when studying root biology is the multicellular complexity of plant roots. For example, -omic analysis at the level of a complex organ such as the root represents an average of the responses of the different cells composing the sample. Consequently, cell specific transcripts, proteins and metabolites as well as cell-specific epigenomic changes will not be revealed resulting in a partial understanding of the specific response of a cell or cell type to a stress and difficulties to fully integrate the various -omic data sets.

To demonstrate that a single cell type model represents an attractive alternative to overcome plant multicellular complexity and to better understand gene networks, we compared the transcriptomes of the soybean root hair to that of the whole root (Libault et al., 2010b). Of the 5671 transcription factor (TF) genes known in soybean (Schmutz et al., 2010; Wang et al., 2010), we were able to detect transcripts for 3960 TF genes mining the whole root transcriptome. Out of the 1711 TFs undetected in the whole root transcriptome, 425 (25%) were only detected in the root hair cell transcriptome. This result is surprising since root hair cells were clearly one of the cell type represented in the root samples used for transcriptomic analysis. We are assuming that the low proportion of root hair cells in the root sample led to a dilution of root hair specific transcripts challenging their detection. This analysis strongly supports the need to work on a single cell type such as the root hair cell rather than an entire tissue to enable a more sensitive and accurate depiction of transcript abundance and, as a consequence, plant cellular responses to environmental perturbation. In addition, working at the single cell level will provide data more amenable to the development of computational models and the mapping of gene networks. Using a single cell type system as a model, the information obtained will be clearly unambiguous and would lead to a better characterization of gene networks.

The understanding of root hair cell biology requires the application of the full repertoire of functional genomic tools. However, major challenges in characterizing the biology of a single differentiated root cell type are the limited access to the root system and the isolation of the root cells of interest. In this manuscript, we describe a method to: (1) homogeneously treat the plant’s root hair cells; (2) easily access the root system and, *a fortiori*, the root hair cell; (3) isolate large quantities of this single cell type.

## LIMITATIONS OF CURRENT TECHNIQUES

The isolation of single differentiated root cell types is limited by: (1) the accessibility to the root system; (2) the cell wall which confers the rigidity of the plant and its overall structure. Laser capture microdissection is a popular technique to isolate specific cells types but it is labor-intensive and cell yields are very limited. Nevertheless, it has been successfully applied to study root biology (Klink et al., 2005; Ithal et al., 2007; Santi and Schmidt, 2008; Takehisa et al., 2012). A second method based on the labeling of cell type by the GFP has been recently established to measure *Arabidopsis thaliana* single plant cell type transcriptomes and their regulation in response to environmental stresses (Zhang et al., 2005; Petersson et al., 2009). Using a collection of transgenic plants expressing the GFP in different root cell lines, *Arabidopsis thaliana* root cell types were isolated after digestion of the cell wall and isolation of the resulting GFP positive protoplasts using cell sorting technology. This strategy allowed the identification of root cell type-specific genes validating the concept of root cell-specific transcriptomes. However, as reported by the authors of these studies, the digestion of the cell wall also led to a few changes of the plant transcriptome independently of the cell line or treatment. In addition, several studies highlighted a massive restructuration of the chromatin and epigenetic marks in leaf protoplasts in comparison to differentiated leaves cells (Zhao et al., 2001; Tessadori et al., 2007; Ondřej et al., 2009; Chupeau et al., 2013). A third method, the INTACT method, was applied on *Arabidopsis thaliana* to isolate hair and non-hair cells and analyze their transcriptome and epigenome (Deal and Henikoff, 2010, 2011). This method is based on the expression of biotinylated nuclear envelope protein under the control of a cell type-specific promoter sequence and the isolation of labeled nuclei using streptavidin-coated magnetic beads. The characterization of a cell-specific promoter is a pre-requisite to the INTACT method. While RNA and chromatin structure can be accessed using the INTACT method, other aspects of the biology of the plant cell such as its entire proteome and metabolome cannot be reached with this method.

**FIGURE 1:**
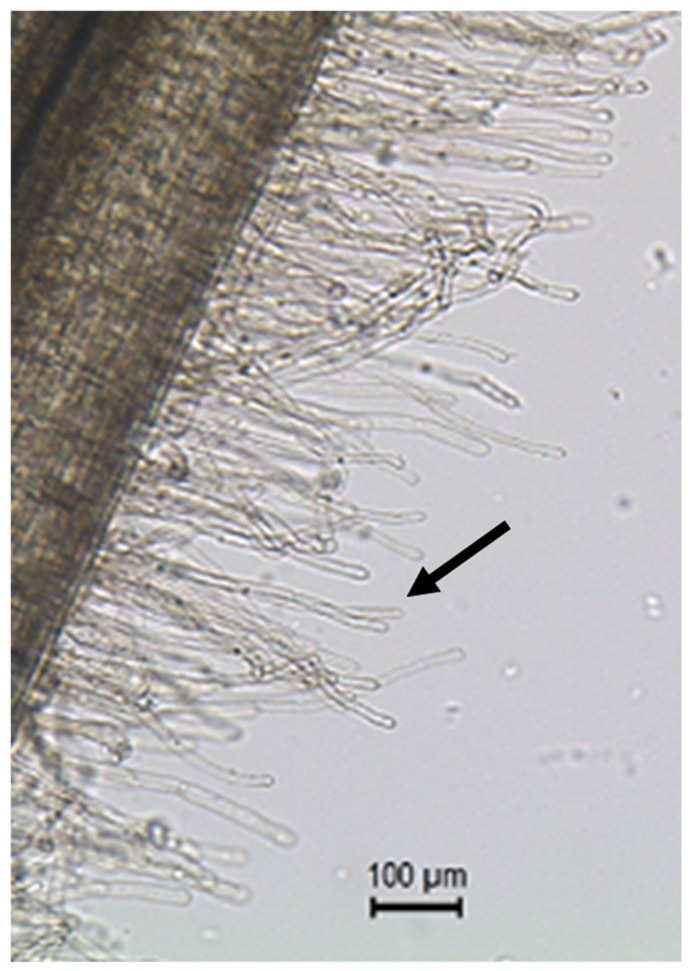
**Root hair cells (black arrow pointing at one of the root hair cells) are single tubular root cells.** Their distinctive lateral elongation increases the surface of exchange between the plant’s root system and the soil. The main function of root hairs is the uptake of water and nutrients from the rhizosphere.

Another strategy to study plant single-cell biology is to massively isolate easily accessible cell types. Such method has been successfully applied on aerial parts of the plant. For example, cotton fiber and pollen cells were isolated to investigate plant cell elongation mechanisms (Franklin-Tong, 1999; Ruan et al., 2001; Arpat et al., 2004; Padmalatha et al., 2012). More recently, the soybean root hair (**Figure 1**) has emerged as a new single cell type model (Libault et al., 2010a). Various studies validate the use of the root hair cell as a model in systems biology through the analysis of the infection of soybean root hair cells by mutualistic symbiotic bacteria [i.e., the soybean root hair cell is the first site of infection by *Bradyrhizobium japonicum*, the nitrogen-fixing symbiotic bacterium involved in soybean nodulation (Gage, 2004; Kathryn et al., 2007)]. In these studies, soybean seedlings were germinated on agar plate preliminary to the inoculation of the plants with *B. japonicum* followed with the isolation of the root hair cells. Various -omics approaches were successfully used to decipher root hair cell biology, including transcriptomic (Libault et al., 2010b), proteomic (Wan et al., 2005; Brechenmacher et al., 2009, 2012), phosphoproteomic (Nguyen et al., 2012) and metabolomic (Brechenmacher et al., 2010) methods. In addition to being a model to investigate plant microbe interactions, the root hair cell is also an excellent model to decipher plant cell regulatory networks in response to abiotic stresses. This is based on their primary role in water and nutrient uptake.

To utilize full potential of this attractive single cell type as a model in root systems biology, root hairs must be evenly treated preliminary to their isolation from the rest of the root system in quantities compatible with any -omic analysis, and a fortiori, transgenic root hair cells must be isolated to perform functional genomic studies at the level of a single cell type. To reach these two goals, we developed the method described below combining the use of an ultrasound aeroponic system to generate and evenly treat a large population of root hair cells and the purification of frozen root hair cells using a highly selective filtration system. This method overcomes the limitations related to the use of the agar media to germinate seedlings such as the heterogeneity of the root hair cell population produced (i.e., root hair cells interact with the agar or are expanding in the atmosphere impacting their physiology) and open new avenues to investigate root hair cell biology because enabling functional genomic studies (see below). To date, we focused on the isolation of soybean root hair cells but the method described below has been validated using other plant models such as maize, sorghum, and rice.

## DETAILED PROTOCOL OF THE OPTIMIZED METHOD

### USE OF AN ULTRASOUND AEROPONIC SYSTEM TO ENHANCE ROOT HAIR DENSITY AND TREATMENT

The study of root hair cell response to stresses presupposes:

1. The even treatment of the root system under control and stressed conditions to minimize biological variations;
2. The optimization of the growth conditions of the root system and the enhancement of the differentiation of root hair cells on the root system;
3. An easy access to the root hair cell compatible with their observation and isolation;
4. The development of methods to efficiently isolate them.

**FIGURE 2:**
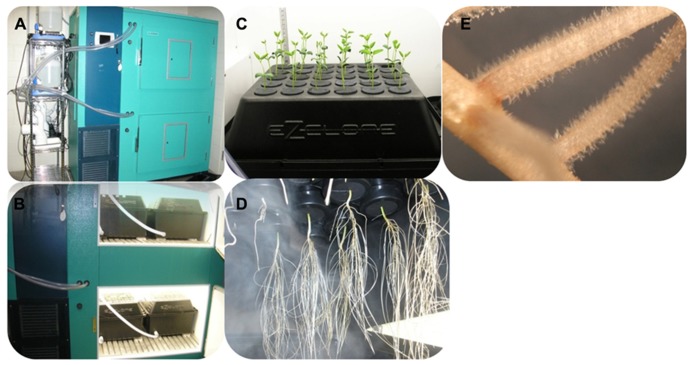
**Soybean seedlings grown in the ultrasound aeroponic system; (A,B) the whole system for plant culturing; (C,D) the plants in the EZ-cloner; (E) soybean root showing a high density in root hair cells**.

We recently developed a method which fulfills these different requirements. Five days-old soybean seedlings germinated on a mixture of vermiculite and perlite (3:1) were transferred to the ultrasound aeroponic system under controlled conditions (long day conditions, 25–27°C, 80% humidity; **Figure 2**). This system is composed of two units: the fogger system and the cloner unit (EZ-CLONE Enterprises Inc.). The fogger system relays on the production of a 5 micrometres (μm) droplets of nutritive solution by ultrasound misters (OCEAN MIST^®^, DK24) which atomize nutrient solution into a nutrient-rich mist by vibrating at an ultrasonic frequency [in the case of soybean, we are using the B&D nutritive solution (Broughton and Dilworth, 1971)]. An air flow pushes the cool mist into the cloner unit where plants are growing. The quantity of mist produced by the fogger system is controlled by the number of mist makers used per fogger system as well as by a timer controlling the frequency and duration of the production of mist. Using a thin mist to feed the plant maximized the oxygenation of the root system, an important factor contributing to a higher density in root hair cells of the root system [(Shiao and Doran, 2000); **Figures 2C,D**]. Altogether, this unique system optimizes root growth, enhances root hair cell density and offers an easy access to the root hair cell compatible with their observation and isolation (**Figure 2E**).

### ROOT HAIR ISOLATION PROCEDURE

Root hair cell isolation has been repetitively applied on soybean (Wan et al., 2005; Brechenmacher et al., 2010; Libault et al., 2010b; Nguyen et al., 2012). Concomitantly to the development of the aeroponic system, the method used to isolate soybean root hair cell was updated to reach two objectives: (1) maintain or enhance the level of purity of the root hair cell preparation from the rest of the root system; (2) maximize root hair yields. Several methods exist to isolate root hairs including gentle brushing of the frozen root system into liquid nitrogen (Bisseling and Ramos Escribano, 2003) or stirring of the roots immersed in the liquid nitrogen with glass rod preliminary to their isolation (Roehm and Werner, 1987; Bucher et al., 1997). The first method maximizes root hair purification but root hair yields are low and the method is labor intensive. The second method provides large quantities of plant material but the root hair cell preparation could be easily contaminated by non-root hair cells such as root fragments resulting from the stirring.

**FIGURE 3:**
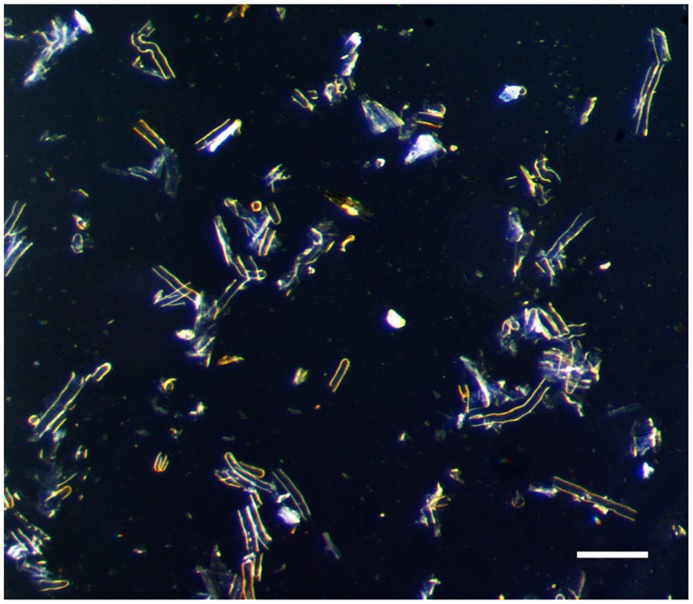
**Isolated root hairs in light microscope.** Bar = 100 μm.

We optimized the latter method as described below. Briefly, the root systems of 3 weeks-old soybean plants are isolated, rapidly wiped off to remove extra moisture then immediately immersed into liquid nitrogen. This rapid freezing prevents undesirable stress of the root and root hair cells due to their manipulation. All subsequent steps are performed in liquid nitrogen. Frozen roots are gently stirred into liquid nitrogen by a glass rod for 10 min. The flow of liquid nitrogen is sufficient to break root and root hairs. The liquid nitrogen containing the root hairs is filtered through 90 μm sieve into a beaker. Based on stereomicroscopic observations, this mesh offers the best compromise to maximize the level of purification of the root hair cells without compromising the yield (**Figure 3**). The stripped roots are rinsed 5–7 times to collect the remaining root hair cells and increase the yield (i.e., as much as 1000 mg of isolated root hair cells were isolated from 63-week old soybean plants). The plant material harvested is usable the most up-to-date molecular approaches.

### MOLECULAR QUANTIFICATION OF THE LEVEL OF PURITY OF THE ROOT HAIR CELL PREPARATIONS

**FIGURE 4:**
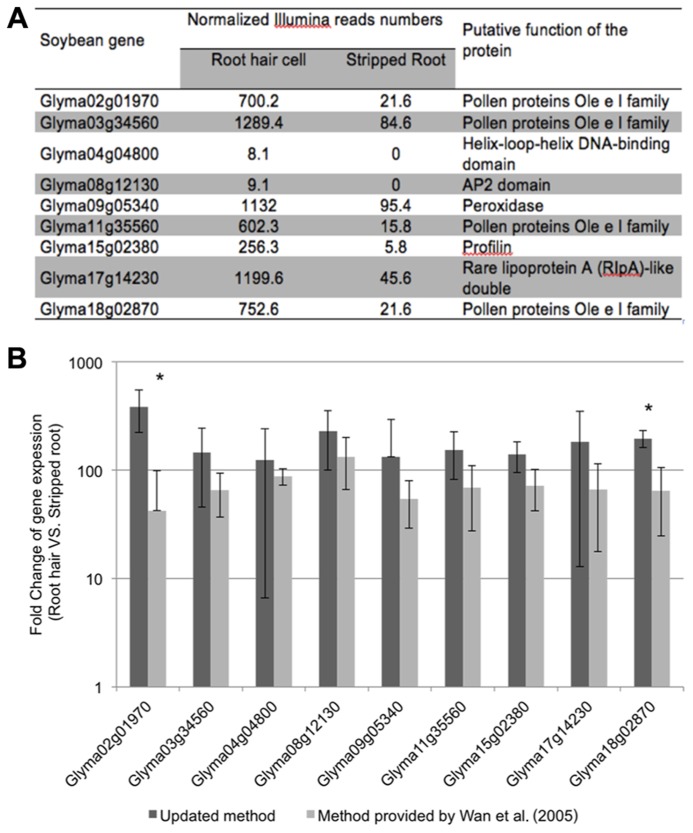
**Expression analyses of soybean root hair specific genes.** (**A**) Relative expression levels of nine soybean genes in root hair cells and stripped roots from the Illumina read data (Libault et al., 2010c); (**B**) The fold-change of the expression of nine root hair specific genes was quantified between isolated root hairs and stripped roots. The plant material was generated using our optimized protocol (dark bars) and the method provided by Wan et al. (2005) (light gray bars). For each experiment, a minimum of three biological replicates were performed and analyzed. The student *t*-test was applied to highlight significant differences between these two methods. The asterisk indicates significantly difference (*p < 0.05).

To evaluate the purity of the root hair cell preparations, we quantified the expression of several “root hair-specific” genes in both isolated root hair and stripped root samples. These genes were selected from the soybean transcriptome atlas (Libault et al., 2010c) based on their high or specific expression in root hair cells compared to stripped roots (**Figure 4A**). We are assuming that the low transcript abundance of these “root hair-specific” genes in stripped roots is the consequence of the presence of remaining root hair cells or root hair cell nuclei in the stripped root samples (i.e., the nucleus of mature root hairs are located in the base of the cell).

The fold change of gene expression level in root hair cell *versus* stripped root ranged from 11.9 (Glyma09g05340) to 44.1 (Glyma15g02380) based on RNA-seq data (**Figure 4A**). Applying qRT-PCR methods, we analyzed the quality of the plant material collected using our optimized method compared to a previous root hair cell isolation method (Wan et al., 2005; **Figure 4B**). Independently of the root hair isolation method used, we observed a higher abundance of transcripts encoded by the nine candidate genes in isolated root hairs compared to stripped roots supporting high levels of purification of the root hair cells. Using root hair cells and stripped roots collected using the method described by Wan et al. (2005), the fold changes of expression of root hair specific genes between root hairs and stripped roots ranged between 42.4 ± 56.6 (Glyma02g01970) and 133.0 ± 67.1 (Glyma08g12130). Our optimized root hair cell method repetitively led to fold changes of expression of root hair specific genes ranging between 124.4 ± 117.8 (Glyma04g04800) and 385.8 ± 164.0 (Glyma02g01970). This result supports a higher enrichment in root hair cells in root hair cell preparation using the updated method compared to Wan et al. (2005) method.

### APPLICATIONS OF THE ULTRASOUND AEROPONIC SYSTEM TO INVESTIGATE BIOLOGICAL QUESTIONS

The flexibility of the use of the ultrasound aeroponic system is fully compatible with the homogeneous treatment of the root system to analyze root hair response to biotic and abiotic stresses. The nature of the abiotic stresses allowed by the aeroponic system is diverse including: (1) changes of the chemical composition of the nutritive solution to analyze root hair response to nutrient deprivation, low and high pHs, salinity or heavy metal contaminations, etc.; (2) changes of the environmental conditions such as temperature, water potential, etc.; (3) inoculation of plants with pathogenic and symbiotic microorganisms. The latter was validated by inoculating 2 weeks-old root systems of the hypernodulation soybean mutants [i.e., NOD1-3, NOD2-4, and NOD3-7; Ito et al., 2007] with a bacterial suspension of *B. japonicum*, the soybean nitrogen-fixing symbiont. As soon as 10 days after inoculation, nodules emerged. Thirty days after inoculation, a large number of nodules were developing on the hypernodulating soybean roots [NOD1-3 (106.8 ± 27.7 nodules per plant), NOD2-4 (159.7 ± 42.7 nodules per plant), NOD3-7 (99.7 ± 29 nodules per plant)]. Compared to these mutants, 4.4- to 7-fold fewer nodules were counted on wild type root system (22.8 ± 9.25 nodules per plant). Although, we found that the number of nodules per root system is lower in the aeroponic system grown plants compared to vermiculite grown plants (i.e., wild type, NOD1-3, NOD2-4, and NOD3-7 mutants showed 67, 441, 344, and 143 nodules per plant, 17–18 days after inoculation, respectively; Ito et al., 2007), the NOD hypernodulating phenotype is observed in the aeroponic system. These data support that this technology is fully compatible with the analysis of the early and late stages of legume nodulation. We assume that additional experiments and tests using the aeroponic system would maximize the number of nodules per plant.

**FIGURE 5:**
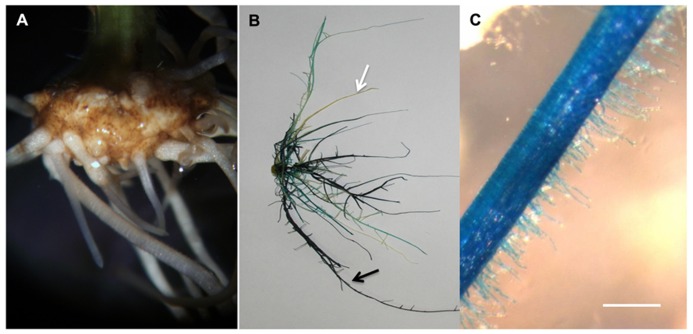
**Soybean transgenic roots and root hairs generated in the ultrasound areoponic system; (A) transgenic roots emerging from the callus 10 days after *Agrobacterium rhizogenes* inoculation; (B) *GUS*-stained soybean root system, the black arrows point at the transgenic root and the white arrows point at the non-transgenic root; (C) *GUS*-stained transgenic root hair cells.** Bar = 200 μm.

Another potential attractive application of the aeroponic system is the generation of composite plants (i.e., plants carrying a mixture of transgenic and non-transgenic roots growing from a wild type shoot) and, *a fortiori*, the easy access to a large mass of transgenic roots compatible with their observation and various molecular analyses. To test this potential utilization of the aeroponic system, we inoculated soybean shoots with *Agrobacterium rhizogenes* carrying our transgene of interest (in this case, a fusion between the cassava vein mosaic virus promoter and the *UidA* gene which encodes the beta-glucuronidase). Ten days after bacteria inoculation, a callus was formed and roots started to emerge (**Figure 5A**). Four weeks after inoculation, the emerged root system was stained using X-Gluc to reveal the β-glucuronisase activity (Libault et al., 2010d; **Figure 5B**). In average, we observed seven transgenic roots emerging from each composite plant. Stereomicroscopic observations revealed that these roots carry an impressive number of transgenic root hair cells (**Figure 5C**).

## CONCLUSION

In this manuscript, we combined the use of an ultrasound aeroponic system with updated method to isolate root hair cells to maximize the potential of plant root hair cell as a single cell type model for systems biology. This updated method has the following advantages: (1) enhance root hair cell density on the root system; (2) even and long-term treatment of the entire population of root hair cells to access the molecular response of the root hairs to various biotic and abiotic stresses.; (3) compatibility with the microscopic observation of the root hair cells; (4) leading to high yields of isolated root hair cells compatible with any -omic analyses.

In addition to be well-suited to perform –omics analyses at the level of one single cell type, the ultrasound aeroponic system has been validated to study plant-bacteria interactions and to produce large quantities of easy accessible plant material allowing functional genomic studies. Undoubtly, our updated method of generating large amount of pure root hair cells will promote the progress of deciphering the regulatory mechanism of plant cell biology including plant cell response to environmental stresses.

## EXPERIMENT PROCEDURE

### PLANT GROWTH

Soybean seeds (*Glycine max *[L.]) were surface-sterilized by three sequential treatments with 1.65% sodium hypochlorite (10 min each), rinsed three times with deionized water before a 10 min treatment with 10 mM hydroxychloride. Seed were finally washed three times with sterile water before sowing on sterilized mixture of vermiculite and perlite (3:1 ratio). Seeds were germinated under permanent light conditions at 25°C. One week later, the seedlings were transferred into fogger system and supplement with the mist of B&D plus 10 mM KNO_3_. Cultured for another 2 weeks in areoponic system, the seedlings were collected into liquid nitrogen for root hair isolation.

### RNA EXTRACTION AND cDNA SYNTHESIS

Total RNAs were extracted using Trizol Reagent (Invitrogen). Six to ten μg of total RNA were extracted from one preparation of isolated root hairs. Total RNA samples were treated with the TURBO DNase (Ambion) according to the protocol provided by the manufacturer before to reverse transcribe 1 μg of DNA-free RNA using oligodT and the Moloney murine leukemia virus reverse transcriptase as previously described (Libault et al., 2010b).

### QUANTITATIVE REAL-TIME PCR AND DATA ANALYSIS

Quantitative real-time PCR (qPCR) primers were designed using Primer3 software^1^ **Table 1**.

**Table 1:** qRT-PCR primers.

Soybean gene ID	Forward primer	Reverse primer
Glyma02g01970	TGGCTGCAAAGTGAAAATGA	TCAATTCTTCGTGCCAATGA
Glyma03g34560	ATGAGTTGGGGCAGTACGAC	TAGTTGAGCTTGACGCCAGA
Glyma04g04800	CCAACGGAACAAAGGTTGAT	TATCGGAGCGTACATCCACA
Glyma08g12130	GCCCAACAAAGGATTAACGA	TATCCTCCACATGGCACTCA
Glyma09g05340	GGCATGACAAGGGCTCATAC	GCCTGTTCCGTTGTTGT
Glyma11g35560	TGCTACGTGAAGCCTGTT	AGTGGAGCACCATTGAGA
Glyma15g02380	CAAGGTGAACCTGGAGCTGT	TCTCCCAACCTCTCAACGAT
Glyma17g14230	CGTGATGAATGTTGGAGGTG	GTTGCAAATGCCTGGTATGA
Glyma18g02870	GACCCTTAGCTTTCCGTCCT	TCTCAATGCATGGTCAAAGG

Quantitative real-time PCR reactions were performed as described by Libault et al. (2008) including an initial denaturation step of 3 min at 95 °C followed by 39 cycles of 10 s at 95 °C and 30 s at 55°C. Dissociation curves were obtained using a thermal melting profile performed after the last PCR cycle: a constant increase in the temperature between 65 and 95°C.

Cycle threshold (Ct) values were obtained based on amplicon fluorescence thresholds. According to Vandesompele et al. (2002), delta Ct were generated using the geometric mean of the cycle threshold of three reference genes [*Cons6*, *Cons7*, and *Cons15* genes Libault et al., 2008]. PCR efficiency (*P*_eff_) for each sample was calculated using LinRegPCR (Ramakers et al., 2003), and the expression level (*E*) were calculated using the equation *E* = *P*_eff(-Δ*Ct*)_. The fold change of the gene expression levels between root hair *versus* stripped root was calculated for each root hair specific gene. Three independent biological replicates were generated for each condition and Student *t*-tests with two tails and two samples equal variance were applied to display the significant differences of gene expression between root hair and stripped root samples. *P* value < 0.05 was regarded significant.

### CLONING AND SOYBEAN HAIRY ROOT TRANSFORMATION

As described by Libault et al. (2010c), cloning of the cassava vein mosaic virus promoter upstream of the *UidA* gene was performed using the Gateway^®^ system (Invitrogen^2^). The cassava vein mosaic virus promoter fragment was introduced first into the pDONR-Zeo vector (Invitrogen) using the Gateway^®^ system BP Clonase^®^II enzyme mix, then into pYXT1 destination vectors carrying the *UidA* genes, using the Gateway^®^LR Clonase^®^II enzyme mix.

Two weeks-old soybean plants grown on pro-mix were used to generate composite plants. K599 *Agrobacterium rhizogenes* bacterial strain carrying the transgene of interest, a transcriptional fusion between the cassava vein mosaic virus promoter and the *UidA* gene, was grown at 30°C in LB medium supplemented with kanamycin. The bacteria were pelleted by centrifugation, and re-suspended in B&D medium supplemented with 10 mM potassium nitrate and acetosyringone (20 μM) to an optical density at 600 nm = 0.35.

Soybean shoots were cut between the first true leaves and the first trifoliate leaf and placed into rock-wall cubes (Fibrgro). Each shoot was inoculated with 4 mL of *Agrobacterium rhizogenes* suspension and then allowed to dry for approximately 3 days (23°C, 50% humidity, long-day conditions) before watering with deionized water. After 1 week, instead to transfer the composite plants into vermiculite-perlite as described by Libault et al. (2009), the transformed soybean shoot were transferred into the ultrasound aeroponic system supplemented with B&D medium plus 10 mM potassium nitrate. After 2 weeks, the β-glucuronisase activity of the soybean root system was revealed as described by Libault et al. (2010d).

## Conflict of Interest Statement

The authors declare that the research was conducted in the absence of any commercial or financial relationships that could be construed as a potential conflict of interest.

## Footnotes

1

2
